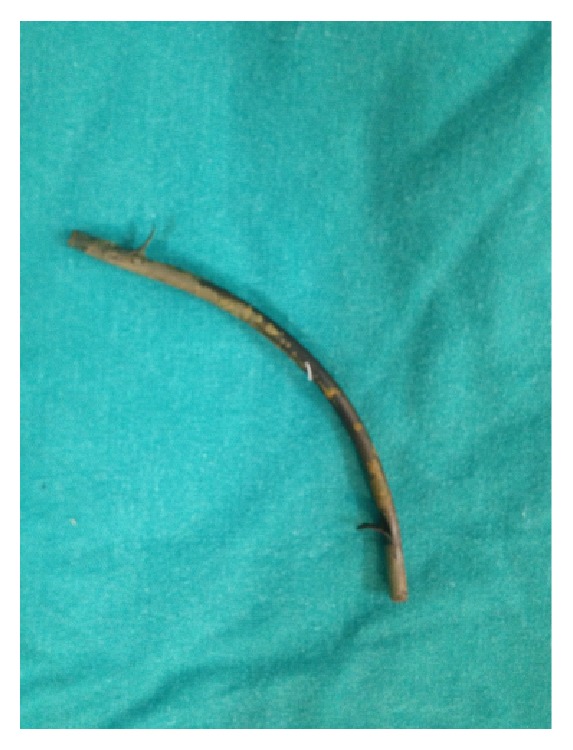# A Rare Complication of Biliary Stent Migration: Small Bowel Perforation in a Patient with Incisional Hernia

**DOI:** 10.1155/2015/860286

**Published:** 2015-07-26

**Authors:** Özkan Yilmaz, Remzi Kiziltan, Oktay Aydin, Vedat Bayrak, Çetin Kotan

**Affiliations:** ^1^Department of General Surgery, Faculty of Medicine, Yüzüncü Yıl University, Van, Turkey; ^2^Department of General Surgery, Faculty of Medicine, Kırıkkale University, Kırıkkale, Turkey; ^3^Department of General Surgery, Ceyhan Government Hospital, Adana, Turkey

## Abstract

Endoscopic biliary stents have been recently applied with increasing frequency as a palliative and curable method in several benign and malignant diseases. As a reminder, although most of the migrated stents pass through the intestinal tract without symptoms, a small portion can lead to complications. Herein, we present a case of intestinal perforation caused by a biliary stent in the hernia of a patient with a rarely encountered incarcerated incisional hernia.

## 1. Introduction

Endoscopic biliary stents have been recently applied with increasing frequency for several benign and malignant diseases. This procedure presents short-term complications such as hemorrhage, pancreatitis, cholangitis, and perforation, in addition to long-term complications such as stent migration and late perforation [1]. In this case report, we present a case of intestinal perforation caused by a biliary stent in the hernia of a patient with a rarely encountered incarcerated incisional hernia.

## 2. Case Presentation

**Figure 1:**
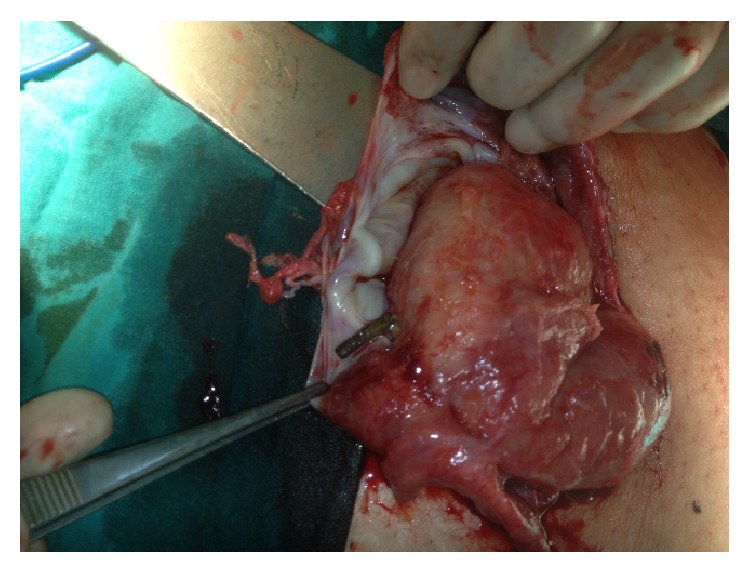


**Figure 2:**
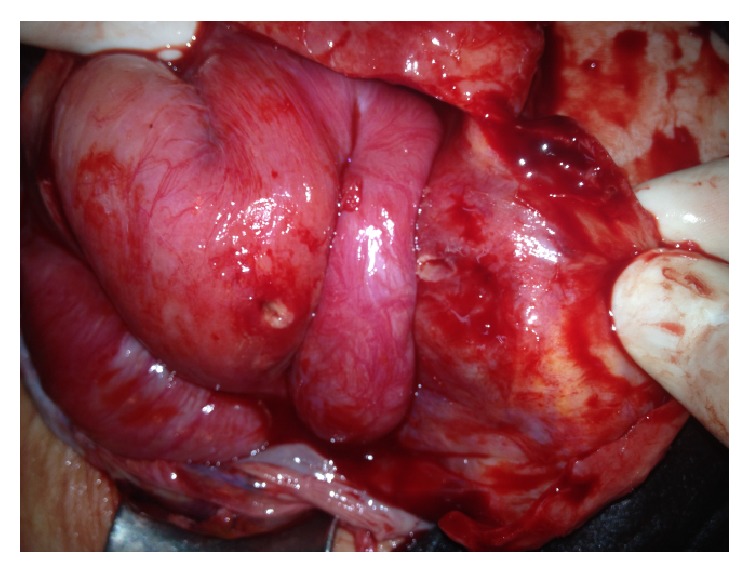


A 52-year-old female patient was admitted to the emergency department with complaints of abdominal pain and the inability to pass gas and stools for two days. The patient received endoscopic retrograde cholangiopancreatography (ERCP) and a biliary stent procedure three years prior due to choledocholithiasis, after which she was surgically treated with a median incision due to a cyst in the liver one and a half years prior. The ERCP was repeated and the stent was replaced due to cholestasis symptoms three months prior to the current presentation. Upon inspection of the patient, a painful-upon-palpation, 10 cm in diameter, irreducible hernia sac with an erythematous surface was palpable, which had herniated from the 4 cm fascial defect at the bottom of the patient's midline incision. Upon discovery of edematous bowel loops and 10 × 5 cm of septal fluid collection surrounding them in the hernia sac during ultrasonography (USG), the patient was taken in for emergency operation. When the hernia sac was opened during the operation, a jejunum loop wrapped with omentum was observed (Figure 1). There were two round perforations in this loop, which were 0.4 cm in diameter and 10 cm apart (Figure 2). The biliary stent that had moved from the proximal perforation was detected and removed (Figure 3). After the primary repair of the perforations, the fascial defect was closed with an overlap. The patient was discharged free of problems on the seventh postoperative day.

## 3. Discussion

Many treatment methods are applied today to malignant or benign biliary strictures. Procedures that rely on percutaneous transhepatic or endoscopic methods are preferred more frequently than surgical interventions. In malignant cases in particular, morbidity rates, such as perioperative mortality and anastomotic stricture, are quite high. The fact that endoscopic and percutaneous biliary interventions are relatively less invasive methods ensures that they are more commonly preferred [2–5].

The transpapillary placement of plastic stents was first described by Soehendra and Reynders-Frederix [6] in 1979 and its use continues to increase today. The rates of complications arising from endobiliary stents have been reported to be between 8 and 10%, mortality rates 1%, and distal migrations up to 6% [5, 7, 8]. The migration rate in plastic stents has been reported to be higher compared to metal stents [9]. It has been reported that the risk of migration is higher in stents placed due to benign causes compared to those placed due to malignant causes [10, 11]. The reason for the more frequent occurrences of stent migration in benign diseases is explained by the greater growth in diameter of the biliary tract in relation to benign causes and the rapid decrease in inflammation after the stent. In malignant diseases, however, the migration rate is reported to be low due to stent fixation resulting from tumor growth [1, 10, 11]. While proximal migration of the stent has been associated with malignant strictures and stents that are wide and short, distal migration has been linked to benign strictures and ampullary stenosis [1]. Some authors suggest that, in order to reduce the risk of stent migration, rather than a single large stent, multiple smaller stents be placed instead [12]. Routine sphincterotomy during biliary stenting is not recommended due to the sphincter of Oddi tonus and valves may help in preventing distal migration [1].

The most frequently encountered problem with endoscopically placed biliary stents is that of restenosis, due to benign or malignant causes [13]. The proximal or distal migration of the stent, on the other hand, is not very common [9]. It is reported that these stents, which undergo migration, are often expelled through natural means or remain in the intestinal tract without causing symptoms [1].

Although the intestinal migration of the choledochal stent is not uncommon, the extralumination of the stent within the intestinal lumen through the intestinal wall is a rare complication. Because it is fixed and “C” shaped, the duodenum has been reported to be the location where stents most frequently extraluminate [14, 15]. In addition, reasons such as adhesions that lead to diversion of the linear course of the intestinal tract, diverticula, and the formation of an intestinal protrusion into the hernia sac have also been reported as conditions that increase the likelihood of stent extralumination. Cases of colovesical fistulae [16], colovaginal fistulae [15], colocutaneous fistulae [17], small bowel perforations [18], and perforations within parastomal hernia [19] have been reported as a result of these pathologies causing extralumination. In the featured case, as well, the stent must have passed into the hernia sac from the protruding intestinal loops through the stages of wall contact, decubitus, perforation, and extralumination most likely due to the diversion of the lumen [20, 21].

We concluded that the stent, which was moving along smoothly within the intestinal lumen, was blocked from following its natural intestinal course when an intestinal “kinking,” caused by reasons such as a hernia, diverticulitis, or adhesions, as in the case of our patient, or luminal pathologies, created a resistance to this thrust, after which the stent, left between these two forces, perforated the intestinal wall.

Consequently, endoscopic biliary instrumentation has been performed with increasing frequency recently, and, due to increased experience and improved technology, the number of patients who undergo a stent procedure is also increasing day by day. Accordingly, there is also a natural rise in complications associated with stents. Biliary stent migrations generally tend to be asymptomatic, despite being common. In patients with a history of a biliary stent that has been placed for any reason and the presence of acute abdominal symptoms, especially if a hernia is present, one must consider the possibility of intestinal perforations. Abdominal X-rays and CT scans of the abdomen may aid in showing the stent in an ectopic location.

## Figures and Tables

**Figure 3:**